# Processed animal proteins in poultry nutrition: nutritional value, digestibility, safety, and applications

**DOI:** 10.1007/s11250-026-05193-5

**Published:** 2026-06-26

**Authors:** M. Naeem

**Affiliations:** Department of Poultry Science, Auburn University, Auburn, AL 36849 USA

**Keywords:** Processed animal proteins, Poultry nutrition, Digestibility, Amino acid availability, Feed safety

## Abstract

Processed animal proteins (PAPs) are concentrated protein ingredients derived from animal by-products through rendering or hydrolytic processes and are increasingly reconsidered in poultry nutrition due to their nutritional and sustainability advantages. This review provides a comprehensive evaluation of the composition, digestibility, functional properties, safety, and practical applications of PAPs in poultry feeding systems. Nutritional value varies considerably depending on raw material origin and processing conditions, with excessive heat treatment reducing amino acid availability through Maillard reactions and racemization. Blood-derived products and spray-dried plasma exhibit high digestibility and functional benefits, particularly in early-life feeding, whereas hydrolyzed feather meal and poultry by-product meal show variable performance depending on processing quality and inclusion levels. Emerging sources such as insect meals and fermented protein hydrolysates offer promising alternatives but require standardization to ensure consistent nutritional value and safety. Digestibility assessment methods differ substantially, and comparisons across studies require careful interpretation to avoid misleading conclusions. Production trials indicate that partial replacement of soybean meal with non-ruminant PAPs can maintain or improve growth performance, gut health, and nutrient efficiency when diets are formulated on a digestible amino acid basis. Safety considerations, including microbial hazards, chemical residues, and regulatory compliance, remain critical and are supported by advances in analytical detection technologies. Overall, PAPs represent a viable and sustainable protein source in poultry nutrition, provided that processing, quality control, and formulation strategies are carefully managed.

## Introduction

To understand the present role of processed animal proteins in poultry nutrition, it helps to trace both the practical drivers behind their use and the regulatory and scientific developments that have shaped what is acceptable in feeds. PAPs are concentrated protein ingredients obtained from animal by-products, slaughterhouse offal, and other materials that are not used for human food (EFPRA 2025). Historically, these materials have been used locally in mixed rations for livestock. In the modern era, processing technologies, rendering, hydrothermal treatment, enzyme hydrolysis, and spray drying, among others, have enabled recovery of protein fractions with improved shelf life, palatability, and nutrient density, and these methods created the ingredient class we now term PAPs.

From a poultry nutrition perspective, the attraction of PAPs is straightforward. Poultry are monogastric animals that require highly digestible protein and a correct balance of essential amino acids (Adhikari et al. 2025). Conventional vegetable proteins such as soybean meal are widely used, but they have limitations: concentrations of certain essential amino acids like methionine may be low, anti-nutritional factors can reduce digestibility unless removed or processed, and global reliance on soybean imports raises both cost and sustainability concerns. PAPs, by contrast, typically contain high levels of crude protein and a better complement of sulfur amino acids and other limiting amino acids in poultry diets, making them attractive complements or partial replacements for plant protein sources (Janmohammadi et al. 2009; Ryvak et al. 2020). Several economic and environmental analyses show that reintroducing PAPs into compound feed can substantially reduce dependence on imported soy and lower feed costs while providing a route to valorize slaughterhouse waste (Veldkamp et al. 2010; Veldkamp 2012; Nath et al. 2023; Lee et al. 2025a). The potential substitution volumes reported for some European contexts are substantial, which is why policymakers and industry stakeholders have taken a careful, evidence-based approach to any changes to feed rules (Veldkamp 2012).

The regulatory story must be told in parallel with the nutritional one because it has directly affected research priorities and industry practice. The bovine spongiform encephalopathy (BSE) crisis and related public health concerns triggered far-reaching bans and restrictions on feeding animal-origin proteins to farm animals in many jurisdictions. In the European Union, a comprehensive set of measures introduced in the early 2000s effectively banned many forms of PAPs in certain food animal feeds, and required strict categorization of animal by-products to protect public and animal health (Fumière et al. 2009). Scientific and analytical advances have progressively allowed regulators to differentiate between ruminant and non-ruminant proteins and to target measures more precisely. The resulting regulatory framework permits the controlled use of non-ruminant PAPs in certain feeds, subject to traceability and processing requirements, as well as robust monitoring to prevent cross-species recycling that could spread transmissible agents (Fumière et al. 2009; Adkin et al. 2011). This cautious regulatory opening has catalyzed a wave of research addressing nutritional performance, processing effects, detection technologies, and risk assessments.

From a scientific standpoint, the practical question underpinning the PAP debate is whether these ingredients can reliably supply digestible amino acids and energy in a way that supports growth, welfare, and product quality in poultry, without introducing unacceptable safety risks. The literature shows nuanced and source-specific answers. Blood products and spray-dried animal plasma, for example, are consistently identified as highly digestible with functional properties that support early-life chick performance and disease resilience; they are rich in immunoglobulins, bioactive peptides, and readily absorbed amino acids (Oba et al. 2019; Kazimierska and Biel 2023). Poultry by-product meals (PBM) and meat and bone meals (MBM) generally provide a concentrated and economical source of protein, minerals, and fat, but their nutritive value varies with raw material composition and processing intensity (Okanovic et al. 2009; Janmohammadi et al. 2009). Feather meal (FM), a keratinous product, historically posed digestibility challenges because keratin is resistant to enzymatic breakdown; however, hydrothermal treatments and enzymatic or hydrolytic processing can transform feather keratin into hydrolysates with dramatically improved digestibility, making FM an increasingly viable PAP when processed appropriately (Fisinin et al. 2019; Zinoviev et al. 2022).

A recurring technical theme is that processing both enables and limits PAP value. Rendering or heat treatments reduce microbial hazards and extend shelf life, but high temperature and long dwell times can degrade amino acids, lead to Maillard reactions, and even racemize specific residues such as aspartic acid. Racemization from L- to D-forms, particularly D-aspartic acid, is a sensitive indicator of severe processing and correlates with reductions in the biological availability of affected amino acids (Bellagamba et al. 2015). The practical consequence is that not all PAPs are equal for nutritionists: two batches labeled as “poultry by-product meal” can differ markedly in digestible amino acid profile and ileal digestibility depending on raw material mix and thermal history. Analytical work evaluating amino acid digestibility, pepsin digestibility, and true metabolizable energy (TME) using precise assays such as the cecectomized precision-fed rooster model has thus been central to establishing realistic feed values for PAPs (Oba et al. 2019).

The reintroduction debate has also stimulated applied production trials. Wageningen Livestock Research and other groups have reported that replacing portions of soybean meal with pig-origin or poultry-origin PAPs can maintain broiler performance and health when diets are balanced for digestible amino acids and mineral contributions (Van Krimpen et al. 2018). Likewise, research on reproductive performance and welfare indicators suggests that PAP inclusion, when properly formulated, does not increase welfare problems and, in some cases, may reduce feather pecking and cannibalism, possibly via improved nutrient balance and satiety (Veldkamp et al. 2010). These findings are not uniform across studies, so the consensus is conditional rather than absolute: PAPs can be nutritionally valuable but must be used intelligently within integrated feed formulation strategies.

Beyond conventional PAPs derived from terrestrial slaughter by-products, a wave of novel PAP sources has emerged in the past decade. Insect meals, notably black soldier fly larvae (BSFL), mealworm, and others, are now regulated in some regions as PAPs and present favorable crude protein concentrations combined with efficient circular production profiles and lower greenhouse gas footprints (Józefiak et al. 2016; Belhadj Slimen et al. 2023). Insect PAPs bring their own technical issues: variability in amino acid profile depending on feed substrate; potential microbiological hazards such as *Clostridium* species; and the need to standardize processing protocols to ensure consistent digestibility (Grenda et al. 2021; Kryuchkov and Nikisov 2025). Fermented proteins from meat processing wastewater and microbial synthesis proteins have also been explored as PAP-like ingredients, showing promising growth and feed efficiency responses in experimental work (Andrianova and Yegorov 2022; Simmons et al. 2023).

Detection, traceability, and contamination control form a third pillar of the PAPs literature because regulatory permission is tightly linked to the capacity to demonstrate species origin, processing class, and absence of prohibited ruminant material. Multiple analytical approaches have been developed and validated. Microscopy remains a workhorse for the simple detection of animal particles and has a low detection threshold for certain morphological structures. Polymerase chain reaction (PCR)-based DNA detection offers species specificity for many matrices, but can be hampered by DNA degradation in heavily processed products and by false positives when milk or other permitted ingredients contribute trace DNA (Fumière et al. 2009; Axmann et al. 2015). Immunoassays and mass spectrometry-based peptide marker approaches have improved tissue and species specificity, enabling reliable detection of ruminant-specific peptides down to regulatory thresholds (Marbaix et al. 2016; Steinhilber et al. 2019). Near-infrared spectroscopy and Fourier transform infrared methods provide rapid fingerprinting that can classify PAP types based on fat or mineral signatures, with strong predictive performance in well-calibrated systems (De La Haba et al. 2009; Pu et al. 2014). The fall-out of this analytical work is that monitoring systems can be designed with complementary methods to satisfy both regulatory demands and industry quality assurance.

Safety considerations are not limited to transmissible spongiform encephalopathy. Recent surveillance work has documented chemical residues such as tetracycline antibiotics in significant proportions of PAP samples, raising questions about residual antimicrobial activity after processing and the potential implications for antimicrobial exposure via feed (Morello et al. 2021). Microbial hazards are also variable in PAPs. For example, insect PAPs may harbor spore-forming *Clostridium* species under some processing regimes, which require targeted risk assessment and mitigation (Grenda et al. 2021). These findings underscore the need to pair nutritional evaluation with rigorous hazard analysis and critical control point approaches in PAP production.

Finally, practical implementation must focus on feed formulation. Nutritionists increasingly depend on digestible amino acid databases and TME values rather than crude protein alone. This is especially vital with PAPs because processing alters digestibility and amino acid availability. Tools and models that enable formulators to include PAPs while meeting digestible lysine and methionine standards, calcium and phosphorus balances, and pellet quality requirements are essential for achieving consistent performance. The research indicates that, when properly processed, analyzed, and formulated, PAPs can serve as reliable protein sources in poultry diets. However, success relies on careful raw material selection, controlled processing, standardized analytical methods, and vigilant safety monitoring. These themes will be expanded in the upcoming sections covering specific PAP types, processing effects, digestibility evidence, production trial results, detection technologies, safety and regulatory frameworks, and practical feed formulation recommendations.

In this context, the present review focuses on PAPs derived from terrestrial animal by-products and emerging alternative sources, with particular emphasis on their composition, digestibility, nutritional variability, safety considerations, and practical implications for poultry nutrition. Although fish meal is excluded from the primary scope of this review, it is occasionally referenced as a benchmark protein source to facilitate comparative evaluation of nutritional value and performance outcomes. By narrowing the scope in this way, this review aims to provide a focused and critical synthesis of current evidence on the re-evaluation and strategic use of non-ruminant processed animal proteins in poultry feeding systems, particularly in light of sustainability pressures, regulatory developments, and advances in processing and analytical technologies. Furthermore, this review was conducted as a narrative synthesis of the literature. Relevant studies were identified through searches in major scientific databases, including Web of Science, Scopus, PubMed, and Google Scholar. The search covered publications primarily from 2000 to 2025, with emphasis on recent developments. Keywords included combinations of “processed animal proteins,” “poultry nutrition,” “digestibility,” “animal by-products,” “insect meal,” and “feed safety.” Studies were selected based on relevance to poultry feeding, nutritional evaluation, processing effects, safety, and regulatory aspects. Both experimental and review articles were included. Non-peer-reviewed sources and studies lacking methodological clarity were excluded. This review does not follow systematic review protocols such as PRISMA and is intended as a narrative synthesis of current knowledge. In this review, the term ‘processed animal proteins (PAPs)’ is used as the primary descriptor for protein ingredients derived from animal by-products through rendering, hydrolysis, or related processing technologies. Other terms, such as animal by-products or animal-derived protein ingredients, are used only where contextually appropriate but are considered within the broader PAP classification.

## Composition of PAPs and the influence of processing on nutritional quality

Understanding the nutritional value of processed animal proteins for poultry requires a close look at both their chemical composition and the degree to which processing conditions shape nutrient availability. PAPs are not uniform commodities; they are families of ingredients whose nutrient profiles depend heavily on the species of origin, the tissue fractions included, the production technologies applied, and the quality control systems operating in the rendering or hydrolysis facility (Axmann et al. 2015). Variability can be wide, and this variability directly informs both formulation decisions and biological outcomes in poultry feeding. Researchers, therefore, tend to frame PAP quality in terms of three connected pillars: composition, processing intensity, and resulting digestibility.

The starting point is composition, which is strongly tied to the raw materials that enter the rendering stream. PBM, for example, may include carcass trim, necks, backs, viscera, and a range of soft tissues. If the material comes from eviscerated carcasses with limited bone, the resulting meal may be high in protein and relatively low in ash. If it includes significant bone content, the ash and mineral fractions increase, diluting protein concentration and shifting phosphorus and calcium values upward (Janmohammadi et al. 2009). A similar story applies to MBM from mixed terrestrial species. Even within a single rendering plant, day-to-day shifts in incoming material can create measurable changes in crude protein, fat, and ash contents. Because poultry diets are sensitive to digestible amino acid supply, this variability matters: a batch with slightly higher ash may still meet crude protein specifications but deliver fewer digestible amino acids per kilogram simply because the protein fraction is proportionally smaller.

Within PAPs of single-tissue origin, such as blood meal (BM) or hydrolyzed feather meal (HFM), compositional characteristics are more predictable but still influenced by upstream handling and processing. BM and spray-dried blood or plasma products are generally recognized for very high crude protein contents and essential amino acid density. Lysine, leucine, and valine levels are notably strong, and in the case of plasma proteins (PPs), functional fractions like immunoglobulins and biologically active peptides provide more than basic nutrient value (Kazimierska and Biel 2023). FM presents a contrasting picture: crude protein values often exceed those of meat meals, but the native keratin matrix is resistant to digestive enzymes, making raw feather protein poorly available to birds. This is where processing becomes central. Hydrothermal treatment, pressure cooking, and enzyme-assisted hydrolysis can disrupt keratin’s disulfide bonds and convert the protein into shorter peptides and free amino acids that are far more digestible. Studies have demonstrated that properly processed HFM can support growth effectively, while under-processed material performs poorly in digestibility assays (Fisinin et al. 2019; Zinoviev et al. 2022).

Processing, in fact, is one of the dominant determinants of PAP quality. The rendering process involves heat, pressure, moisture reduction, and fat separation, and these steps can either preserve or degrade amino acids. If the temperature or residence time is excessive, several alterations occur. Maillard reactions between reducing sugars and amino groups can reduce lysine availability. Oxidation can affect sulfur amino acids, and extreme thermal loads can cause racemization of amino acids such as aspartic acid, producing D-isomers that are often biologically less available. Bellagamba et al. (2015) demonstrated that D-aspartic acid concentration can serve as a sensitive processing marker because the shift from L- to D-forms is strongly associated with intense heat treatments. When racemization is extensive, digestible amino acid content in the final product tends to drop, underscoring why thermal histories matter for true nutritional value.

The picture is similar for MBM, where excessive temperatures can reduce peptide solubility and increase cross-linking between proteins, lowering digestibility even when crude protein numbers appear stable. The poultry nutrition community, therefore, places significant emphasis on measuring standardized ileal digestibility (SID) or TME, rather than relying on proximate composition alone. Oba et al. (2019) illustrated this point clearly in their cecectomized rooster assay work. When comparing chicken-based ingredients processed under different conditions, they observed substantial differences in amino acid digestibility and metabolizable energy, even though proximate analysis suggested only modest differences. These findings validate the use of precision-fed assays and digestibility coefficients in feed formulation, rather than assuming uniformity across similarly named PAP ingredients.

Heat influence is not solely negative. Proper rendering is essential for microbial safety and fat stabilization, and controlled thermal application can create beneficial hydrolysis of collagen and connective tissues. But the window between sufficient processing and overprocessing is narrow. Rendering plants manage this by calibrating cookers, monitoring moisture levels, testing particle sizes, and using in-process temperature sensors. Quality assurance programs that track these parameters reduce variability and improve the predictability of nutrient profiles.

Beyond traditional rendering, several alternative processing technologies produce high-quality PAPs with distinctive nutritional attributes. Spray-drying, widely used for blood plasma and BM, preserves functional proteins effectively because drying is rapid and thermal exposure is relatively mild compared with batch rendering. The result is a protein ingredient with excellent solubility, minimal amino acid damage, and high digestibility. PPs, in particular, retain immunoglobulins and bioactive peptides that are implicated in improved gut function and early chick performance (Kazimierska and Biel 2023). In hydrolyzed proteins from poultry or mixed by-products, enzyme-assisted hydrolysis yields peptide-rich powders with good solubility and digestibility. Such hydrolysates have been studied not only as poultry feed ingredients but also as biofertilizers and specialized aquaculture feed additives because of their rapid absorption and functional bioactivity (Lee et al. 2025b).

Novel PAPs such as insect meals add another layer of complexity because processing methods vary substantially across producers. BSFL meals, for example, can be defatted, full-fat, extruded, or minimally heated. Each approach yields different crude protein values, fatty acid profiles, and digestibility outcomes. Józefiak et al. (2016) noted high microbiological quality in many insect-based PAPs but also pointed to the need for consistent processing standards to ensure stable amino acid profiles. More recent work has underlined that variations in rearing substrate, thermal treatment, and drying method can shift both nutrient density and microbial risk (Grenda et al. 2021). Enzyme hydrolysis of insect biomasses, studied by Kryuchkov and Nikisov (2025), demonstrates how controlled hydrolytic treatment can further enhance digestibility and produce low molecular weight peptides. Yet, the rapid evolution of insect processing technologies means values must be checked for each supplier rather than assumed based on generic ingredient descriptions.

Fermentation-based PAPs, although less common, show promise as well. Simmons et al. (2023) examined protein hydrolysates recovered from meat processing wastewater via microbial fermentation. These hydrolysates contain peptides with varying chain lengths and favorable amino acid balances. Feeding trials revealed that broilers receiving diets containing these fermented hydrolysates exhibited strong feed efficiency and weight gain responses, suggesting that peptide-rich PAP alternatives can perform competitively with conventional protein sources. Microbial single-cell proteins and other substrate-derived hydrolysates fall conceptually under the PAP umbrella, where animal-origin substrates are used, and similar themes of processing influence and digestibility variation apply (Andrianova and Yegorov 2022).

Even when composition and processing produce a nutritionally strong product, purity and the absence of undesirable residues influence practical feeding decisions. Studies investigating antibiotic residues in PAPs highlight the importance of processing on chemical stability. Morello et al. (2021) detected tetracycline residues in a sizable minority of tested PAP samples, noting that even in rendered meals, these compounds exhibited residual antimicrobial activity. This suggests that processing may not fully denature or deactivate certain chemical contaminants, and that monitoring programs must look beyond pathogenic hazards. Likewise, protein oxidation markers can inform whether extended storage or inadequate fat separation has compromised protein integrity and, by extension, nutritional value.

Analytical characterization of PAPs often utilizes both classical chemical methods and modern spectroscopic or proteomic techniques because composition alone does not capture the full nutritional story. Near-infrared and mid-infrared spectroscopy, for instance, can classify PAPs rapidly by predicting fat content, ash content, or species origin based on unique spectral fingerprints (De La Haba et al. 2009; Pu et al. 2014). These tools help nutritionists and regulators verify that products match declared specifications and have not undergone adulteration or unintended cross-contamination. Proteomic profiling has become particularly important in identifying tissue-specific peptide markers, allowing researchers to quantify the presence of certain tissue types and better understand the biological availability of the protein fraction (Marbaix et al. 2016; Steinhilber et al. 2019). These strength-in-detection approaches complement nutrient assays because the distribution of tissues in a PAP, such as collagen-rich versus muscle-rich fractions, directly affects amino acid balance and digestibility.

The intersection of composition and processing is most evident when comparing digestibility outcomes across PAP types. Studies illustrate this by linking chemical characteristics with true digestibility values. For example, Oba et al. (2019) reported that chicken-based ingredients with limited processing damage showed high TME and excellent digestible amino acid supply, whereas more heavily processed variants dropped markedly in biological value. Janmohammadi et al. (2009) similarly documented that PBM with moderate ash and well-controlled rendering parameters exhibited suitable nutritive value for broiler feeding, but batches with excessive ash or thermal exposure exhibited reduced performance.

Taken as a whole, the literature paints a consistent picture: the nutritional value of PAPs lies not only in their baseline composition but also in how well that composition is preserved through processing. The more predictable the process, the more predictable the digestible nutrient delivery in poultry diets. When processing minimizes amino acid degradation, maintains peptide solubility, and ensures microbial safety, PAPs can deliver high-quality protein with digestibility comparable to or even surpassing many plant proteins. But when processing is excessive or inconsistent, the apparent crude protein becomes a misleading indicator of actual nutritional potential.

As poultry nutrition increasingly emphasizes digestible amino acids, energy efficiency, and sustainability metrics, PAPs can support these goals but only if their compositional and processing variations are well understood and effectively managed. Future research is expected to refine processing technologies to reduce amino acid losses, improve peptide release from connective tissue proteins, standardize insect meal production, and enhance rapid analytical tools for assessing real-time nutritional quality. The interplay of composition and processing will therefore remain central to both the scientific evaluation and commercial use of PAPs in poultry feeding.

## Digestibility, amino acid availability, and metabolizable energy of PAPs for poultry

Evaluating the nutritional value of processed animal proteins requires more than describing their chemical composition. For poultry, what ultimately matters is the fraction of nutrients that can be digested, absorbed, and used for growth, maintenance, immune function, and production. Digestibility and metabolizable energy, therefore, sit at the center of any assessment of PAP quality. They reflect how processing, raw material composition, and protein structure affect the biological usability of amino acids and energy. Across the literature, digestibility emerges not only as a measurement but as a narrative thread running through all evaluations of PAPs, from blood products to feather hydrolysates and novel insect meals.

The starting point for digestibility evaluation is recognizing that PAPs, unlike many plant proteins, contain a wide spectrum of digestible and indigestible fractions, depending on the tissues involved. Muscle-rich sources such as PBM typically show higher digestibility of indispensable amino acids than collagen-rich sources, while blood-derived proteins often show digestibility values approaching the upper limits achievable in commercial feeds. PPs, in particular, maintain excellent digestibility when processing preserves the integrity of their functional protein fractions (Kazimierska and Biel 2023). Much of their nutritional strength arises from their solubility and the absence of fibrous protein structures that resist enzymatic hydrolysis. Studies consistently report high apparent ileal digestibility (AID) of lysine, leucine, valine, and other essential amino acids in spray-dried plasma (SDP) and BM, which is partly why blood products remain valued in the early diets of chicks, where high nutrient availability supports sensitive early-stage physiology.

In contrast, the digestibility picture for collagen-rich or keratin-rich materials is more complicated. FM, composed largely of keratin, shows low native digestibility due to the stability of keratin’s disulfide cross-links. Early research showed that untreated feathers offered little usable protein value. However, hydrothermal and enzymatic processing fundamentally changed this narrative. Zinoviev et al. (2022) and later Fisinin et al. (2019) documented that carefully controlled hydrolysis, whether through short-term high-pressure treatments or enzymatic cleavage, disrupts keratin’s structural rigidity and increases peptide length distribution, enabling substantial improvements in apparent digestibility and broiler performance. Their work demonstrates that the digestibility of FM is not an inherent limitation but a processing problem, and when solved, feather-derived PAPs can contribute usefully to poultry diets.

It is important to distinguish between different digestibility and energy evaluation systems when interpreting PAP data. AID does not account for endogenous amino acid losses and may underestimate true nutrient availability, whereas SID corrects for basal endogenous losses and provides a more accurate measure for formulation. TME, commonly determined using precision-fed rooster assays, reflects energy availability but is not directly comparable with ileal amino acid digestibility metrics. Therefore, caution is required when comparing studies using different methodologies, as direct equivalence may lead to misleading conclusions. This methodological distinction is particularly relevant when comparing digestibility values across different PAP sources and processing conditions discussed in the following sections.

The methodological framework used to measure digestibility influences how these ingredients are assessed. Precision-fed cecectomized rooster assays, highlighted in Oba et al. (2019), remain one of the most sensitive tools for evaluating digestible amino acids and TME. In their comparison of chicken-based ingredients processed under different conditions, Oba and colleagues showed that even ingredients with similar proximate analyses delivered markedly different digestible amino acid concentrations and TME values. Over-processed meals consistently exhibited lower digestibility, confirming the link between thermal history and nutrient availability. The study also confirmed that crude protein alone is a poor predictor of biological value. Instead, digestible amino acids, particularly digestible lysine, are the limiting factors that nutritionists must calibrate when incorporating PAPs into poultry diets.

Amino acid availability is tightly tied to processing intensity. Excessive heat can cause Maillard reactions that reduce lysine availability, alter sulfur amino acids, and shift aspartic acid from its natural L-form to the D-form. Bellagamba et al. (2015) emphasized racemization as an integrative marker of processing severity. When high proportions of D-aspartic acid appear in a PAP, it signals that protein structure has undergone irreversible thermal damage, a finding that reliably correlates with reductions in digestible amino acid content. Their work supports the broader consensus that PAPs should be evaluated not only chemically but also structurally, since amino acid integrity determines the actual nutrient supply to birds.

MBM and PBM typically exhibit moderate to high digestibility when processing is controlled. Janmohammadi et al. (2009) reported that PBM with balanced ash content and adequate but not excessive rendering temperatures provided digestible amino acid profiles suitable for broiler feeding. Ash content is not trivial. Ingredients with excessively high bone content dilute protein and introduce mineral loads that can interfere with digestive enzyme activity, reduce palatability, and ultimately decrease the efficiency with which amino acids are absorbed. Therefore, meal composition interacts with processing to shape true nutrient availability.

Novel PAPs derived from insects contribute a different digestibility profile. Józefiak et al. (2016) observed that insect meals, particularly BSFL, often show moderate to high protein digestibility but must be evaluated on a species-specific and process-specific basis. Differences in drying temperature, defatting, and substrate composition can shift both protein structure and digestibility outcomes. Grenda et al. (2021) further showed that microbial hazards and substrate-driven compositional differences complicate the interpretation of digestibility findings, making clear that standardizing production is essential before broad conclusions can be drawn. Insect protein hydrolysates, as studied by Kryuchkov and Nikisov (2025), illustrates how targeted hydrolysis can increase digestibility by releasing shorter peptides, similar to the improvements seen in FM processing. Still, insect PAPs occupy a developing area where digestibility data will continue to expand and standardize.

Hydrolysis-based PAPs from meat industry by-products also show strong digestibility potential. Simmons et al. (2023) reported that fermented protein hydrolysates recovered from wastewater streams exhibited high solubility and excellent digestibility in feeding trials, supporting robust feed efficiency and weight gain in broilers. The presence of short-chain peptides likely facilitates rapid absorption, offering a functional advantage compared with intact protein sources that require extensive enzymatic digestion. Similar observations apply to single-cell proteins examined by Andrianova and Yegorov (2022), though these ingredients sit at the boundaries of conventional PAP classification.

Energy availability forms the second half of the nutritional picture. TME values for PAPs vary according to fat content, degree of hydrolysis, and the proximity of protein structure to digestible forms. PBM generally show moderate energy contributions due to residual fat content and the digestibility of muscle proteins. FM, once hydrolyzed, may contribute more modest energy because of their lower fat levels but compensate with concentrated amino acids. BMs and plasma products, despite their exceptional amino acid digestibility, can present moderate TME values because they are low in fat. Thus, formulating diets with PAPs requires balancing protein contribution with energy density, often pairing PAP inclusion with adjustments to added fats or energy-rich ingredients.

Anti-nutritional influences are rarely primary concerns in PAPs compared with plant proteins, yet processing defects can produce indirect anti-nutritional effects. Oxidative damage to lipids and proteins can reduce nutrient density, and residues from antibacterial drugs, as documented by Morello et al. (2021), may interfere subtly with microbiota-driven digestion. While such issues are not digestibility phenomena in the classical sense, they modify the digestive environment and thus the effective nutrient supply to birds.

A consistent conclusion across the literature is that digestibility is not simply an inherent property of PAPs but a function of their processing history. Renderers and feed manufacturers who control thermal exposure, particle size, moisture removal, and enzymatic treatment can achieve PAPs with strong and predictable digestibility profiles. Conversely, inconsistent processing translates into inconsistent biological outcomes, which is why regulators and industry professionals closely monitor processing parameters.

The implications for poultry nutrition are clear. When digestibility and metabolizable energy values are properly characterized and integrated into formulations based on digestible amino acids rather than crude protein, PAPs can serve as reliable and often highly effective contributors to balanced diets. Their digestibility advantages, particularly in blood-derived and hydrolyzed protein products, offer strategic benefits in early feeding programs and in stages where amino acid efficiency is critical. As sustainability concerns and economic pressures intensify, PAPs that deliver high digestible nutrient density offer a route to reducing dependence on imported plant proteins without compromising performance. Yet success depends on understanding the digestibility implications of each PAP type and applying rigorous quality control from production to formulation.

## Performance outcomes, health effects, and welfare implications of PAPs in poultry feeding

The practical test of any feed ingredient lies in how birds perform on it. Poultry respond not only to nutrient density but also to the digestibility, palatability, and functional bioactivity of ingredients. PAPs show a spectrum of effects on growth performance, feed intake, carcass quality, and bird health. Consistently, these outcomes reflect the same forces discussed in the earlier sections: composition, digestibility, processing quality, and ingredient type. When those elements align, PAPs deliver performance comparable to or better than vegetable proteins; when they do not, variability in growth responses emerges.

### Poultry by-product meal

PBM is increasingly recognized as a promising alternative protein source in animal feeding systems, driven by sustainability concerns and the rising cost of conventional ingredients such as fishmeal. Research indicates that its influence on growth performance is not uniform, but rather depends on inclusion level, processing method, and the use of dietary supplements. In broiler chickens, Vithana et al. (2023) reported that replacing conventional protein sources with PBM did not significantly alter body weight or feed conversion ratio, even when diets were supplemented with exogenous protease. Although protease inclusion showed a numerical tendency to improve feed conversion, the lack of statistical significance suggests that enzymatic supplementation alone may not fully overcome the inherent variability in protein quality and amino acid digestibility of PBM. These authors attributed this limitation to inconsistent raw material composition and reduced digestibility of certain amino acids, highlighting a key nutritional constraint of PBM-based diets. Earlier broiler research supports the view that PBM responses are strongly inclusion dependent. However, Mahmood et al. (2018) provided a contrasting perspective, demonstrating that low-level inclusion of PBM can be beneficial. In their study, inclusion of PBM at 3% significantly improved body weight gain and feed efficiency during the starter and grower phases. However, increasing PBM levels beyond this threshold reduced feed intake and did not yield additional gains in body weight, indicating diminishing returns at higher inclusion rates. This suggests that PBM can enhance early growth performance when used sparingly, but higher levels may negatively affect palatability or nutrient utilization. Similarly, Manzoor et al. (2014) evaluated graded replacement of fishmeal with PBM in broiler rations supplemented with amino acids. Their findings showed that PBM inclusion at 2 to 6% significantly improved growth performance compared with an 8% inclusion level. Importantly, feed efficiency and dressing percentage were not adversely affected across treatments, suggesting that appropriate amino acid supplementation can partially mitigate the nutritional shortcomings of PBM. Nevertheless, the decline in performance at higher inclusion levels reinforces concerns regarding amino acid imbalance and digestibility constraints. Sahraei et al. (2012) observed significant differences in body weight gain and feed conversion ratio across grower and finisher phases in broilers fed varying levels of PBM. Their results identified 60 g/kg of diet as the most practical and beneficial inclusion rate, supporting optimal growth and feed efficiency. This finding underscores the importance of identifying species- and phase-specific inclusion thresholds rather than assuming linear benefits with increased PBM levels. Processing quality further affects the nutritional value of PBM. Ahmad et al. (2017) demonstrated that antioxidant-treated PBM could be safely included in broiler diets up to 7% without adverse effects on performance. However, excessive antioxidant application negatively affected feed intake and growth, suggesting that overprocessing may impair nutrient availability or palatability. This highlights the need to carefully manage both ingredient stabilization and processing intensity.

Comparative evaluations of animal by-product meals also place PBM in a moderate position relative to other rendered proteins. Swe et al. (2022) reported that broilers fed fishmeal achieved superior growth and feed conversion compared with those fed PBM, FM, or BM. While PBM performed better than FM and BM, it did not match the growth response observed with fishmeal, indicating that PBM is a functional but not equivalent substitute. Evidence from turkey nutrition further supports cautious inclusion. Awachat et al. (2012) showed that poultry slaughter by-product meal could be incorporated up to 5% in turkey poult diets without negative effects on growth, feed intake, feed efficiency, or immune response. Inclusion levels of 7.5% or higher significantly reduced performance, reinforcing the concept that poultry species tolerate only limited inclusion of by-product meals. The relationship between digestibility and performance emerges repeatedly. Janmohammadi et al. (2009), assessing PBM produced in Iranian rendering systems, reported that broilers fed diets containing optimized levels of PBM exhibited comparable feed intake and weight gain to birds fed soybean meal-based diets. In their study, the best-performing PBM batches were those with moderate ash content and controlled processing temperatures, which preserved amino acid availability. These results mirror the digestibility findings of Oba et al. (2019), who showed that meal quality is heavily dependent on processing history. Where overprocessing had occurred, lower amino acid digestibility corresponded with reduced growth performance, underscoring the sensitivity of poultry growth to variations in digestible lysine and methionine. Critically, these studies collectively demonstrate that PBM is not a uniform ingredient, and its nutritional value is highly context-dependent. While low to moderate inclusion levels can sustain or modestly improve growth performance, higher inclusion rates often lead to reduced feed intake, poorer feed conversion, or inconsistent growth responses. Variability in raw material composition, amino acid imbalance, and processing quality remains the primary constraint. Therefore, PBM should be viewed as a complementary protein source rather than a complete replacement for high-quality conventional proteins, and its use must be supported by careful formulation, amino acid supplementation, and quality control to achieve consistent growth performance in poultry.

Collectively, the evidence indicates that PBM can serve as an effective complementary protein source in poultry diets when inclusion levels are moderate, and diets are formulated on a digestible amino acid basis. Positive responses are most consistently observed at low-to-moderate inclusion rates, where PBM can maintain or modestly improve growth performance and feed efficiency. In contrast, higher inclusion levels frequently result in reduced feed intake, less predictable growth responses, and diminished performance benefits. These inconsistencies appear to be driven largely by variation in raw material composition, ash content, amino acid balance, and processing quality rather than by PBM itself. Consequently, successful utilization of PBM depends on rigorous quality control, appropriate inclusion limits, and, where necessary, the use of amino acid supplementation or exogenous enzymes to optimize nutrient availability. Under these conditions, PBM represents a practical and sustainable alternative that can partially replace conventional protein ingredients without compromising broiler performance.

### Feather meal

FM represents a different narrative: its utility depends entirely on processing quality. Zinoviev et al. (2022) demonstrated that broilers receiving HFM obtained by short-duration hydrothermal processing showed significantly improved performance compared with birds receiving inadequately processed material. The authors attributed this to increased digestibility of sulfur-rich amino acids and release of peptide-bound protein otherwise locked within keratin. Fisinin et al. (2019) found that enzymatically improved feather protein could serve as an economical protein source without reducing growth or feed efficiency. These studies collectively demonstrate that FM is not inherently inferior but conditionally valuable depending on the quality of hydrolysis applied.

Processed PBMs, such as FM, require technological modification to overcome inherent limitations in digestibility and utilization. Yeh et al. (2023) investigated the effects of enzymatically degraded FM and a two-stage fermented FM produced using *Bacillus* subtilis var. natto N21 and *Saccharomyces* cerevisiae Y10 in broiler diets. The authors reported that inclusion of enzymatically degraded FM was tolerated only up to 10%, as higher levels linearly and quadratically depressed weight gain, feed intake, and feed conversion, indicating residual constraints associated with keratin-rich substrates. In contrast, broilers fed the fermented FM exhibited improved weight gain, feed conversion ratio, and production efficiency factor over the control during the starter to finisher period. Enhanced apparent and standardized ileal amino acid digestibility further supported the nutritional advantage of fermentation, although sulfur-containing amino acids and tryptophan remained comparatively less digestible. The absence of differences in carcass traits suggests that the primary benefits of fermentation are expressed through growth efficiency rather than carcass modification. Overall, this study underscores fermentation as a critical processing strategy to improve the feeding value of FM, while also highlighting the need for controlled inclusion levels to avoid performance penalties.

Processing methods such as fermentation appear critical in unlocking the nutritional value of otherwise poorly digestible animal by-products. Iheukwumere et al. (2025) evaluated fermented chicken FM as a sustainable feed ingredient and reported significant improvements in broiler growth performance, with body weight increasing progressively from the starter to the finisher phase. Although the feed conversion ratio fluctuated during early growth, birds fed the fermented FM outperformed the control group during the later growth stages, indicating improved nutrient utilization over time. In addition to performance responses, increased organ weights and favorable shifts in hematological indices, including elevated white and red blood cell counts, suggest enhanced physiological status and immune competence. The reduction in certain leukocyte subpopulations further points to a potentially moderated inflammatory response. Overall, the study supports fermentation as an effective strategy to improve the biological value of FM, positioning fermented poultry feathers as a viable alternative protein source when long-term growth efficiency and bird health are considered.

The nutritional value of FM is strongly determined by the intensity and type of processing applied prior to inclusion in broiler diets. Salehizadeh et al. (2025) evaluated the replacement of soybean meal with FM processed through boiling or hydrolysis using enzymatic or bacterial treatments. The authors reported that substituting up to 33% of soybean meal with HFM did not adversely affect body weight gain or feed conversion in Ross 308 male broilers, demonstrating that adequately hydrolyzed keratin can be efficiently utilized. In contrast, diets containing boiled FM at both 33 and 67% replacement levels, as well as HFM at 67%, significantly reduced growth performance, carcass yield, and nutrient digestibility. These results indicate that while moderate inclusion of properly processed FM can partially replace conventional plant protein sources, excessive inclusion or inadequate processing compromises nutrient availability. The study reinforces the central role of processing technology and inclusion limits in determining the feasibility of FM as a high-performance protein ingredient in broiler nutrition.

Enzymatic treatment has emerged as a practical approach to enhance the feeding value of keratin-rich by-products. Ayanwale et al. (2023) evaluated keratinase-treated and untreated FM in broiler diets and reported significant differences in growth performance, carcass traits, and meat mineral composition among treatments. Keratinase treatment improved the nutritional profile of FM, particularly ash and nitrogen-free extract contents, which translated into better growth responses compared with untreated FM. Importantly, FM-based diets were utilized at inclusion levels of up to 24% without compromising growth performance, highlighting a relatively high tolerance when appropriate enzymatic processing is applied. Meat quality was largely unaffected in terms of sensory attributes, with only appearance and flavor showing treatment-related differences. Overall, the study demonstrates that keratinase-treated FM can function as an effective alternative protein source in broiler diets, provided that enzymatic processing is employed to overcome the inherent limitations of untreated FM.

The inclusion level of FM may also impact the response of broilers. Olabode et al. (2022) found that partial replacement of fishmeal with FM significantly affected growth performance and economic returns. Replacing 25% of fishmeal with FM produced the best biological response, yielding the highest final body weight, average daily gain, and a favorable feed conversion ratio. Whereas moderate inclusion at 50% replacement reduced growth slightly but delivered the highest profit and cost benefit ratio, indicating an economic advantage despite minor performance tradeoffs. In contrast, high inclusion at 75% replacement resulted in the poorest growth and feed efficiency, confirming that excessive reliance on FM compromises nutrient balance and utilization. Overall, the findings suggest that FM can effectively replace a portion of fishmeal in broiler diets, with optimal performance achieved at low to moderate replacement levels, while higher inclusion should be avoided to prevent growth depression.

Recent work has further clarified the limitations and potential of fermented FM in broiler nutrition. Kuo and Wei (2024) evaluated a solid-state fermented FM produced using *Bacillus* velezensis PN1 and reported that a 5% inclusion level did not significantly affect final body weight at 35 days when compared with commercial HFM. Growth performance between the fermented and HFM groups was comparable, particularly in diets without supplemental crystalline amino acids, indicating similar bioavailability of protein from both sources. However, both FM treatments resulted in significantly lower weight gain than diets containing 5% fish meal, highlighting that feather-based proteins remain nutritionally inferior to high-quality animal proteins. Despite this, fermentation markedly improved feather degradation, digestibility, and essential amino acid content relative to raw feathers, demonstrating that microbial processing can enhance the feeding value of FM. These findings suggest that fermented FM can serve as a partial protein source in broiler diets when properly processed, but its inclusion should be carefully balanced and supplemented to avoid growth penalties compared with premium animal protein ingredients. Collectively, the literature demonstrates that the processing method is a stronger determinant of feather meal value than feather meal inclusion per se. Enzymatic hydrolysis and fermentation consistently improve digestibility and bird performance, whereas inadequately processed feather meal frequently depresses growth and nutrient utilization.

### Blood and bone meal

Research has expanded the discussion on unconventional animal-derived protein sources by emphasizing integrated by-product mixtures rather than single ingredients. Shuma et al. (2025) reviewed the use of dried blood rumen content mixtures as sustainable poultry feed ingredients and highlighted their high crude protein content, reported to reach up to 80%, alongside additional fiber contributions from rumen contents. The review suggests that inclusion of dried blood rumen content mixtures at approximately 5 to 10% in broiler diets can enhance growth performance, feed efficiency, and carcass quality, while also offering substantial economic benefits through estimated feed cost reductions of 15 to 30%. However, the authors also identified key constraints, including palatability issues, processing and safety considerations, and limited empirical data defining precise inclusion thresholds. Although not classified strictly as processed animal proteins, the findings reinforce the broader potential of blood-based feed resources when appropriately processed and formulated, while underscoring the need for standardized processing methods and further controlled feeding trials.

Blood-derived PAPs appear prominently in performance literature because of their exceptional digestibility. SDP and BM, when properly processed, support excellent growth in early-life feeding programs. Kazimierska and Biel (2023) describe the functional proteins in plasma, immunoglobulins, bioactive peptides, and soluble proteins that support gut integrity and immune development in young animals. Poultry studies leveraging these attributes often report improved early growth, reduced post-hatch mortality, and better resilience under infectious or environmental stress. Blood-derived PAPs, therefore, offer nutritional and immunomodulatory value that plant proteins cannot easily replicate. The inclusion of BM in poultry diets has been reported to improve growth performance, feed utilization, and carcass characteristics. Research shows that BM is a high-protein ingredient, containing up to 80% crude protein, and can be used as an effective substitute for conventional feed sources such as fish meal and soybean meal, especially in areas where feed availability is limited and costs are high (Salifu et al. 2025; Shuma et al. 2025). Evidence from broiler studies indicates that birds fed diets containing different levels of bovine BM achieved growth rates and feed conversion efficiencies similar to those of birds fed control diets, indicating that BM can replace up to 50% of fish meal without negative effects (Salifu et al. 2025). Furthermore, BM supplementation has been associated with better hematological indices and improved overall health status in poultry, which contributes to enhanced growth performance (Salifu et al. 2025). Despite these benefits, determining appropriate inclusion rates and addressing issues related to palatability remain important considerations that require additional research to fully optimize the use of BM in poultry feeding programs (Maphios et al. 2025; Shuma et al. 2025). Ekugba et al. (2024) evaluated the use of bovine BM in broiler diets and found that a 5% inclusion level significantly improved growth performance and morphometric characteristics in both Ross 308 and Marshall strains. Birds fed the bovine BM-based diet showed higher body weights compared to the control, with Ross 308 recording the highest mean body weight after eight weeks. The study also reported significantly greater weight gain in Ross 308 compared to Marshall and control groups, as well as notable improvements in body length, heart girth, and thigh length, indicating enhanced physical development and overall growth performance.

Efforts to reduce reliance on soybean meal have prompted evaluation of composite animal by-product feeds in finisher broiler diets. Diarra et al. (2023) assessed heat-processed bovine blood rumen digesta meal combined with a vegetable oil concentrate as a partial substitute for soybean meal and reported improved weight gain and feed conversion efficiency in birds fed the test diets compared with the control. Notably, the authors demonstrated that up to 50% of soybean meal could be replaced during the finisher phase without compromising performance. These results suggest that appropriate heat processing and strategic formulation with supplemental energy sources can mitigate the limitations typically associated with blood and rumen-based ingredients, enabling their effective use in high inclusion scenarios during later growth stages. The efficacy of BM as a substitute for plant-based protein sources appears to be influenced by inclusion level and bird genotype. Salifu et al. (2023) evaluated the replacement of soybean meal with dried bovine BM in SASSO birds and demonstrated that graded inclusion up to 10% significantly enhanced growth performance. Birds fed 5% dried bovine BM showed the highest dry matter intake, while increasing inclusion levels improved final body weight and daily weight gain, with the best feed conversion efficiency observed at 10% inclusion (*P* < 0.05). Importantly, all haematological and serum biochemical indices remained within normal physiological ranges, indicating no adverse health effects. These findings suggest that dried bovine BM can serve as an effective and economically viable partial replacement for soybean meal in slow-growing broiler strains when used at appropriate inclusion levels.

The response of broilers to BM inclusion appears to be highly dependent on inclusion level and processing method. Arabi and Adam (2021) investigated the optimum level of sun-dried BM under Sudanese production conditions and reported that moderate inclusion improved growth performance. Diets containing 3 to 5% sun-dried BM resulted in superior body weight gain and feed conversion compared with the control diet, indicating efficient utilization at these levels. However, feed intake declined progressively as BM inclusion increased, suggesting potential palatability constraints at higher levels. Carcass yield and mortality were not affected by dietary treatment, confirming that performance differences were primarily nutritional rather than health-related. Overall, the study supports the strategic use of sun-dried BM at low to moderate inclusion levels, while reinforcing the need for precise formulation to balance intake and growth responses.

The use of processed animal byproducts within precision-formulated diets has gained attention as a strategy to improve nutrient efficiency while reducing environmental impact. Askri et al. (2025) reported MBM inclusion did not compromise body weight, feed conversion ratio, or overall carcass yield. Notably, breast meat yield was significantly improved, indicating a favorable redistribution of protein deposition rather than a general growth response. In addition, the low crude protein formulation markedly reduced nitrogen intake and excretion, underscoring the environmental advantages of integrating processed animal proteins into balanced diets. These findings suggest that animal byproducts such as MBM can play a functional role in sustainable broiler nutrition by maintaining performance, enhancing valuable carcass components, and mitigating nitrogen losses when used within well-designed low-protein feeding strategies. Across studies, blood-derived proteins appear most effective when used as targeted supplements rather than major protein sources. Their benefits are linked to high amino acid digestibility and, in the case of plasma products, functional bioactive compounds that support gut health and immune competence.

### Plasma proteins

Blood plasma–derived proteins, particularly SDP and animal blood plasma powders, have gained increasing attention in poultry nutrition due to their dual nutritional and functional properties. Unlike conventional protein ingredients that primarily supply amino acids, blood PPs are rich in biologically active compounds such as immunoglobulins, bioactive peptides, growth factors, and albumin. These components enable PPs to influence growth performance indirectly through improvements in gut health, immune modulation, and stress resilience, rather than acting solely as a crude protein source. The functional nature of some protein fractions also shapes health outcomes linked to PAPs. PPs, as described by Kazimierska and Biel (2023), contain immunoglobulins that support gut immunity. Their inclusion in early diets often results in a lower incidence of enteric challenges and improved gut morphology. Similarly, peptide-rich hydrolysates created from meat industry wastewater, examined by Simmons et al. (2023), exhibited functional bioactivity that positively influenced gut health and feed efficiency when fed to broilers. These hydrolysates contain short-chain peptides that can be rapidly absorbed and may regulate gut barrier function or immune responses in ways that intact protein sources do not. Animal blood–derived protein supplements have also been examined in processed forms such as plasma, with generally positive responses in broiler production. Alam et al. (2023) evaluated dietary supplementation of animal blood plasma powder and reported significant improvements in growth performance, particularly body weight gain and feed conversion ratio, as supplementation levels increased. In addition to performance benefits, birds receiving blood plasma powder exhibited a higher dressing percentage compared with the control group, indicating enhanced carcass yield. Importantly, these productive gains were achieved without detrimental effects on health status, as mortality rates and antibody titers remained within acceptable ranges. Collectively, the findings suggest that blood plasma powder may offer functional advantages beyond basic protein supply, potentially linked to improved nutrient utilization and immune modulation, thereby supporting its inclusion in broiler diets as a value-added animal protein ingredient.

Functional animal-derived proteins such as plasma have been shown to interact strongly with dietary nutrient density and early life physiology. Bundur et al. (2024) evaluated SDP supplementation in corn-soy-based broiler diets formulated at two levels of digestible amino acids and metabolizable energy. The inclusion of SDP significantly increased feed intake and body weight gain and improved feed conversion during the starter phase, highlighting its particular relevance during early growth when digestive and immune systems are still developing. Changes in organ weights further suggest systemic metabolic effects rather than simple protein substitution. Overall, these findings support SDP as a functional feed ingredient that can enhance early growth performance, especially when integrated into high-quality, nutrient-dense broiler diets. A consistent finding across broiler studies is the positive effect of plasma supplementation on early growth performance. Dabbou et al. (2021) demonstrated that broilers fed spray-dried porcine plasma exhibited significantly higher body weights at 12, 25, and 40 days of age compared with control diets and globin-based protein sources. The reported increase in final body weight, reaching approximately 130 g by day 40, highlights the growth-promoting potential of PPs during critical developmental stages. Importantly, this improvement was accompanied by enhanced protein digestibility, suggesting that PPs may improve nutrient utilization efficiency rather than simply increasing feed intake.

The growth-enhancing effects of PPs appear closely linked to their impact on intestinal integrity and immune function. Şahin et al. (2025) highlighted that SDP supplementation improves gut barrier function, modulates inflammatory responses, and reduces oxidative stress in broilers. These physiological effects likely explain why PPs are particularly effective under suboptimal or challenging conditions. Supporting this, Bregendahl et al. (2005) reported that spray-dried bovine PP significantly improved growth performance and breast meat yield in broilers raised under unsanitary, high-antigen environments, whereas little to no benefit was observed under clean experimental conditions. This context-dependent response suggests that PPs function primarily as health-supporting ingredients rather than universal growth promoters. Feeding strategy and inclusion level are critical factors influencing the efficacy of PPs. Beski et al. (2016) demonstrated that higher inclusion levels and longer feeding durations improved body weight and feed conversion ratio during the starter phase. However, excessive inclusion or prolonged feeding led to reduced nutrient digestibility at later ages, indicating diminishing returns and potential inefficiencies. These findings reinforce the concept that PPs should be strategically applied, particularly during the starter period, rather than used as long-term protein replacements.

Overall, the poultry literature clearly demonstrates that blood plasma and PPs can enhance broiler growth performance, particularly by improving feed efficiency, early body weight gain, and carcass yield. However, these benefits are highly dependent on bird age, health status, environmental conditions, and feeding duration. PPs should not be viewed as direct substitutes for conventional protein meals but rather as functional ingredients that support gut health and immune competence. When strategically incorporated, especially during the starter phase or under production stress, blood PPs represent a valuable tool for improving broiler performance and robustness in modern poultry systems.

### Insect meal

Insect-derived PAPs bring a newer dimension to performance evaluations. Józefiak et al. (2016) highlighted that insect meals generally support good microbiological safety and moderate to high nutrient availability, making them viable alternatives in poultry diets. When used at modest inclusion rates, BSFL meal often yields growth performance patterns similar to soybean meal. However, the literature stresses that insect PAP performance is sensitive to rearing substrate, processing method, and final nutrient profile (Rossi et al. 2025). Grenda et al. (2021) showed that *Clostridium* contamination could appear in inadequately processed insect meals, affecting not only safety but also potentially gut health and feed efficiency. Kryuchkov and Nikisov (2025) reported that enzymatic hydrolysis of insect raw material improved digestibility and subsequently improved feed conversion ratios when included in broiler diets. Such findings show that insect PAPs carry substantial promise, but standardization remains a prerequisite for consistent performance. Khan et al. (2023) evaluated the partial and total replacement of soybean meal with maggot larvae meal in broiler chicken diets. They found that weekly and overall body weight gain, feed intake, and feed conversion ratio were not significantly affected by the replacement diets. The authors reported no significant differences in protein efficiency ratio, dressing percentage, apparent metabolizable energy, antibody titer, or amino acid digestibility among the treatment groups. In addition, organoleptic qualities, intestinal histomorphology, hematological indices, and serum biochemical parameters remained statistically similar across all diets. The study concluded that soybean meal can be completely replaced with maggot meal in broiler diets without negative effects on growth performance, nutrient utilization, or overall health.

Beyond their role as feed ingredients, insect larvae have also been explored as enrichment materials with potential physiological implications. Bongiorno et al. (2022) evaluated the provision of live BSFL to medium growing chickens and found that supplementation did not impair overall growth or slaughter performance, supporting their safe use from a productivity standpoint. However, sex specific differences were evident, as control females achieved a superior feed conversion ratio compared with larvae-supplemented birds, suggesting a possible tradeoff between enrichment-driven feeding behavior and feed efficiency. The longer consumption time observed in females further indicates altered feeding dynamics rather than nutritional limitation. Notably, increased spleen and bursa of Fabricius weights in supplemented birds point to potential immunomodulatory effects, which were supported by changes in blood biochemistry, including reduced gamma-glutamyl transferase activity. Nevertheless, lower leukocyte and monocyte counts in larvae fed groups raise questions regarding the consistency and direction of immune responses. Overall, the study suggests that live BSFL may function more effectively as behavioral and welfare enrichments than as direct nutritional enhancers, with subtle impacts on efficiency and immune indicators that warrant further investigation under commercial feeding conditions.

The effectiveness of insect-derived protein meals appears to depend not only on inclusion level but also on the feeding strategy applied across growth phases. Chen et al. (2025) evaluated Scarabaeiform larvae meal supplementation in yellow feathered broilers and demonstrated that phased inclusion strategies were superior to continuous supplementation. Broilers receiving 4 or 8% larvae meal in a phased manner showed significantly higher body weight and weight gain from 1 to 42 days and maintained improved growth performance during the later growth phase, whereas continuous inclusion at similar levels depressed growth rates (*p* < 0.05). Beyond performance, phased supplementation increased abdominal fat deposition and intramuscular fat content, indicating altered energy partitioning and potential effects on meat quality. Importantly, continuous larvae meal feeding elevated pro inflammatory cytokine levels, while phased inclusion mitigated this immune response, suggesting improved physiological adaptation. Changes in gut microbiota composition, particularly enrichment of *Faecalibacterium* in the 4% phased group, further support a mechanistic link between feeding strategy, metabolic regulation, and immune balance. Collectively, these findings highlight that strategic, phase-specific use of insect meals may maximize growth benefits while avoiding adverse inflammatory and metabolic consequences associated with prolonged exposure. Although results vary among insect species and production systems, most studies suggest that insect meals can partially replace conventional protein sources without compromising growth. However, responses remain highly dependent on processing method, inclusion level, and substrate composition, highlighting the need for greater standardization.

### Mixed PAPs

Performance trials conducted in controlled experimental conditions frequently demonstrate the potential of PAPs to replace soybean meal partially without compromising growth. Van Krimpen et al. (2018) found that broilers receiving diets where soybean meal was partly substituted with non-ruminant PAPs maintained weight gain and feed conversion efficiency when digestible amino acids were balanced correctly. This work emphasized that PAPs should not simply be swapped in based on crude protein values but should be formulated using standardized ileal digestible amino acids, an approach that aligns with the digestibility results presented by Oba et al. (2019). That study showed how chicken-based ingredients processed with minimal thermal damage produced high digestible amino acid values and TME, both of which translated into strong biological performance in feeding systems. Comparative evaluations of animal by-products highlight that not all alternative protein sources exert equivalent effects on broiler performance. Swe et al. (2022) assessed the inclusion of different animal by-products in broiler diets and reported that broiler chickens fed diets containing 5% fishmeal achieved the best growth performance, whereas those receiving 5% BM exhibited the poorest final body weight and feed conversion ratio. The inferior performance associated with BM is attributed to potential amino acid imbalances and reduced palatability, which may have limited feed intake and nutrient utilization, suggesting that, despite its high crude protein content, BM requires careful formulation and possibly complementary protein sources to avoid performance depression when used in broiler diets.

A broader synthesis of feeding trials provides insight into stage-specific responses to specialty protein ingredients in broiler nutrition. Njeri et al. (2024) conducted a meta-analysis examining processed animal proteins, insect proteins, and processed conventional vegetable proteins and demonstrated that processed animal proteins significantly improved average daily gain during the starter and grower phases, with the strongest growth response occurring in the starter period. In parallel, processed animal proteins reduced nitrogen excretion during the grower and finisher phases, although an increase in nitrogen excretion was observed during the starter phase, indicating a temporal shift in nitrogen utilization efficiency. Insect protein included at levels below 10% reduced nitrogen excretion primarily during the starter and grower phases, whereas processed conventional vegetable proteins consistently reduced nitrogen losses across all growth stages. Notably, growth responses differed by protein class and phase, with the greatest average daily gain achieved by insect protein in the starter phase, processed animal proteins in the grower phase, and processed vegetable proteins in the finisher phase. Collectively, this meta-analysis underscores the importance of matching protein source and inclusion level to specific growth stages in order to optimize both performance and nitrogen efficiency in broiler production systems.

Comparative assessments of animal protein sources suggest that performance outcomes are often driven more by associated by-product inclusions than by the protein source itself. Erikanobong (2024) evaluated fishmeal and grasshopper meal as animal protein sources in broiler diets in combination with graded levels of sugarcane scraping rumen content mixture. The study found no significant differences in growth performance between fishmeal and grasshopper meal, indicating comparable nutritive value at the tested inclusion levels. In contrast, increasing the inclusion of the sugarcane scraping rumen content mixture beyond 5% significantly affected growth performance, with performance declining at 10 and 15% inclusion. Broilers achieved optimal performance when either grasshopper meal or fishmeal was combined with a 5% rumen content mixture, emphasizing the importance of limiting fibrous by-product inclusion. These results highlight that while alternative animal proteins can effectively replace conventional sources, the accompanying non-protein fractions largely determine overall diet quality and growth response.

The development of novel processed animal protein additives has further demonstrated the potential of these ingredients to enhance broiler productivity. Saleeva et al. (2021) evaluated new animal-derived protein additives in broiler diets and reported a significant improvement in productive performance relative to control treatments. Birds receiving the optimized formulations achieved an 8.47% increase in average live body weight alongside improved feed conversion efficiency. In addition to growth responses, selected treatments yielded superior meat yield and favorable sensory characteristics, indicating benefits extending beyond simple performance metrics. These findings suggest that advancements in processing technologies can improve the functional value of animal by-products, allowing them to contribute positively to both efficiency and product quality in broiler production systems.

Beyond growth metrics, PAPs influence health and welfare, often in subtle but important ways. Veldkamp et al. (2010) reported no negative impacts on behavior, mortality, or welfare indicators when PAPs replaced vegetable proteins in balanced diets. They noted that in some cases, PAP-inclusive diets even reduced undesirable behaviors such as feather pecking, potentially due to improved amino acid balance or increased satiety from high-protein meals. These observations align with the broader understanding that welfare in poultry is closely tied to nutrient adequacy, especially amino acid balance, which influences feather development, stress resilience, and general activity levels.

At the same time, the literature underscores that health risks can arise if PAPs are not processed or monitored correctly. Antibiotic residues documented by Morello et al. (2021) pose a potential concern not only for antimicrobial resistance but also for disruption of the gut microbial environment. Although the levels detected were variable, the persistence of residual antimicrobial activity in some PAP samples shows that rendering temperatures do not always degrade chemical residues effectively. If such residues alter gut microbiota, they could indirectly influence nutrient absorption and bird health. This makes residue monitoring essential when PAPs are incorporated into poultry diets.

The mineral contribution of PAPs also shapes health outcomes. MBM, for example, supplies highly available phosphorus and calcium, which can help reduce dependence on inorganic mineral supplements. However, if ash content is excessive, mineral load may exceed optimal levels, predisposing birds to imbalances that impair growth, affect skeletal development, or hinder nutrient absorption. Janmohammadi et al. (2009) observed that PBM samples with high ash content produced weaker growth responses compared with more balanced samples, likely due to shifts in calcium-to-phosphorus ratios and the dilution of digestible protein.

Carcass traits provide another dimension of performance. Diets containing PAPs generally produce carcass yields equivalent to those of birds fed vegetable-based diets, provided amino acid balance is maintained. Beyond growth performance, plasma supplementation has been associated with improvements in carcass traits and skeletal development. Bundur et al. (2024) reported increased carcass yield and enhanced tibial strength in broilers fed SDP, without adverse effects on mineral composition. Such outcomes suggest improved nutrient partitioning and overall physiological development, which may contribute to economic benefits beyond live weight gain alone. Van Krimpen et al. (2018) noted comparable breast muscle deposition across treatments in their broiler study. Feather processing studies, such as Zinoviev et al. (2022) and Fisinin et al. (2019) found no negative effects on carcass composition at appropriate inclusion rates of HFM. In some insect meal trials reviewed by Józefiak et al. (2016), modest improvements in thigh meat yield have been observed, though this varies across species of insect and production systems.

Dietary substitution of conventional animal protein sources with insect meals has shown variable outcomes on broiler performance and product quality, highlighting the need to distinguish between growth responses and meat quality attributes. Maphios et al. (2025) evaluated the replacement of BM with mealworm larvae (*Tenebrio molitor*) meal in broiler starter diets and reported no significant effects on growth performance or carcass traits (*p* > 0.05), suggesting that neither protein source conferred a measurable advantage in terms of early growth. However, despite the lack of performance differences, the inclusion of mealworm meal significantly influenced meat quality parameters. Meat tenderness was reduced across all diets containing mealworm meal, and cooked meat color was significantly altered at 50 to 100% replacement levels (*p* < 0.05). These findings indicate that while mealworm larvae meal can effectively replace BM without compromising growth, its impact on sensory and physical meat attributes may present limitations for consumer acceptance and should be carefully considered in practical feeding strategies. Feed intake responses to PAPs can vary. BM and plasma products often improve palatability, whereas some meals high in ash or improperly processed materials may reduce palatability. Variation in palatability corresponds with processing quality; rancidity, oxidation, or burnt aromas from over-rendering can suppress feed intake. These sensory dimensions are rarely discussed in proximate analyses but are noted in practical feeding trials as important contributors to performance variation.

**Fig. 1:**
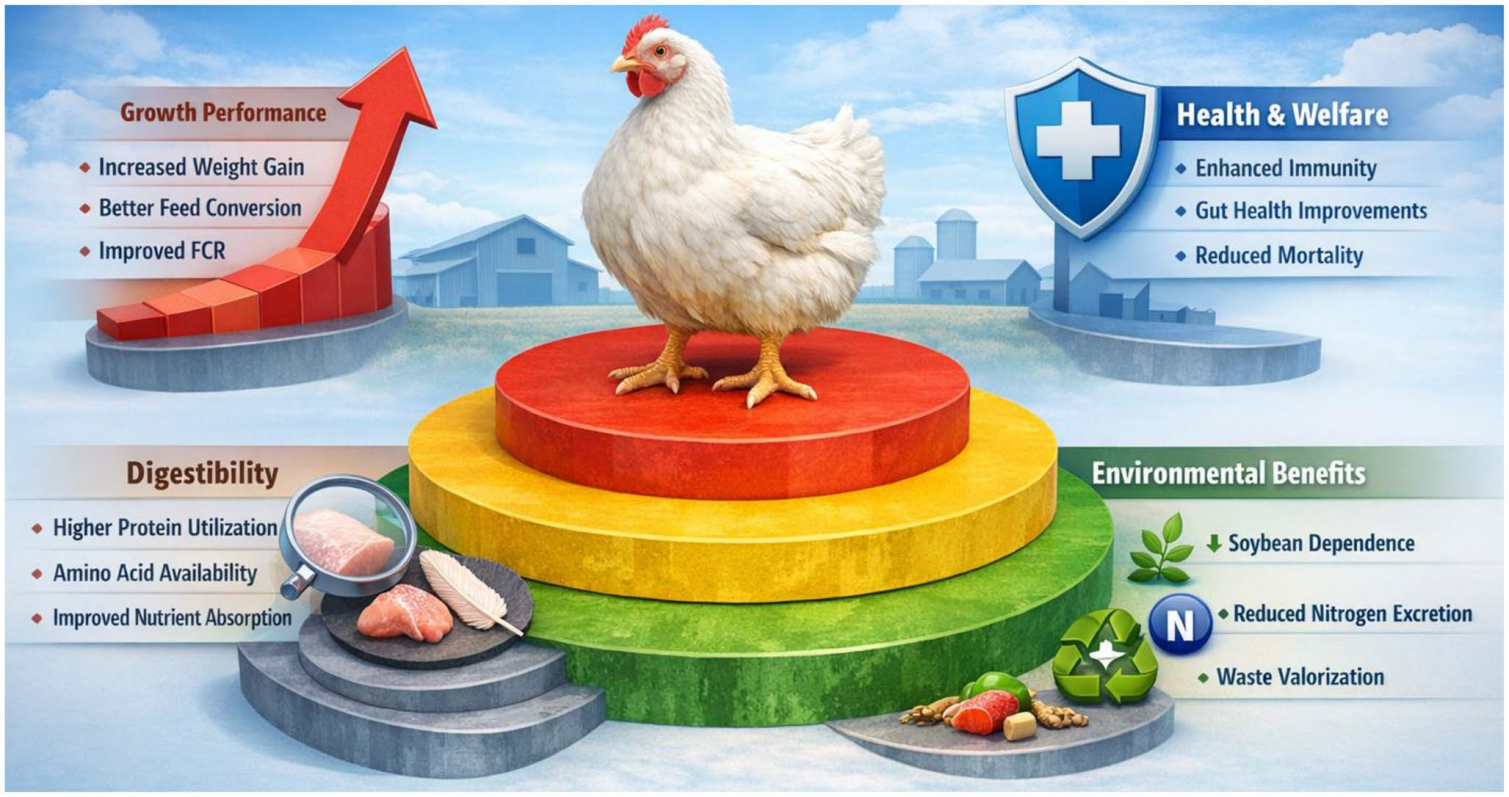
Conceptual illustration of the effects of PAPs on poultry performance, digestibility, health, and sustainability (created with BioRender)

Taken together, in terms of performance, literature reveals a consistent trend: PAPs can function as high-quality protein ingredients that support strong growth, efficient feed conversion, and stable health and welfare outcomes when sourced and processed correctly. The factors that condition their success, digestible amino acids, adequate processing, microbial safety, and residue control, mirror those that determine their digestibility and composition. In this sense, performance outcomes are the integrative reflection of all upstream processes and quality control measures in PAP production. Under well-managed systems, PAPs not only match the performance of conventional plant proteins but may, in specific contexts such as early-life feeding or functional hydrolysate inclusion, provide distinct advantages not easily replicated by vegetable-based ingredients. A summary of key studies examining the use of PAPs in poultry nutrition and their reported impacts on performance, digestibility, and health is given in Table 1, while a conceptual illustration of the effects of PAPs on poultry performance, digestibility, health, and sustainability is shown in Fig. 1.

**Table 1:** Summary of key studies examining the use of PAPs in poultry nutrition and their reported impacts on performance, digestibility, and health

Type of PAP used	Poultry species	Main impact on poultry	Representative Reference
PBM	Broiler chickens	Comparable growth and feed intake to soybean meal when ash content and processing were controlled	(Janmohammadi et al. 2009)
MBM	Broiler chickens	Provided concentrated protein and minerals, performance dependent on processing quality	(Okanovic et al. 2009)
Mixed non-ruminant PAPs	Broilers	Partial replacement of soybean meal maintained growth, welfare, and reduced feather pecking	(Veldkamp et al. 2010)
Non-ruminant PAPs	Poultry (risk assessment)	Demonstrated safety of controlled PAP use under strict traceability systems	(Adkin et al. 2011)
PBM	Broiler chickens	Optimal inclusion around 60 g/kg improved growth and feed conversion	(Sahraei et al. 2012)
PBM	Broilers	Partial replacement of fishmeal improved growth at 2–6% inclusion	(Manzoor et al. 2014)
Heat-processed PAPs	Poultry feed ingredients	Amino acid racemization (D/L aspartic acid) identified as a marker of severe heat processing associated with reduced protein quality	(Bellagamba et al. 2015)
Insect meal (BSFL)	Broiler chickens	Supported normal growth and acceptable digestibility at moderate inclusion	(Józefiak et al. 2016)
SDP	Broilers	Improved early growth and feed efficiency, diminishing returns at later stages	(Beski et al. 2016)
PBM	Broilers	Improved weight gain and feed efficiency at low inclusion (≈ 3%)	(Mahmood et al. 2018)
Mixed non-ruminant PAPs	Broilers	Soybean meal could be partially replaced without performance loss when AA balanced	(Van Krimpen et al. 2018)
HFM	Broilers	Improved digestibility and growth when keratin was adequately hydrolyzed	(Fisinin et al. 2019)
Chicken-based PAPs	Broilers	Processing intensity strongly influenced digestible amino acids and metabolizable energy	(Oba et al. 2019)
Spray-dried porcine plasma	Broilers	Higher body weight and improved protein digestibility	(Dabbou et al. 2021)
Rendered PAPs	Poultry feed safety	Detected tetracycline residues, highlighting the need for routine screening	(Morello et al. 2021)
BM, FM, PBM	Broilers	Fishmeal superior; PBM intermediate; BM lowest performance	(Swe et al. 2022)
Hydrothermally processed FM	Broilers	Significant improvement in sulfur amino acid digestibility and growth	(Zinoviev et al. 2022)
Fermented protein hydrolysates	Broilers	Improved feed efficiency and weight gain due to peptide-rich composition	(Simmons et al. 2023)
Blood PPs	Poultry/livestock	High digestibility, immune support, and improved early-life performance	(Kazimierska and Biel 2023)
PBM	Broilers	No significant change in growth; numerical improvement with protease	(Vithana et al. 2023)
Maggot larvae meal	Broilers	Complete replacement of soybean meal without negative effects	(Khan et al. 2023)
Blood plasma powder	Broilers	Improved weight gain, feed conversion, and dressing percentage	(Alam et al. 2023)
Insect PAPs	Poultry	Identified microbiological risks emphasizing processing control	(Grenda et al. 2021)
Fermented FM	Broilers	Comparable to HFM but inferior to fishmeal	(Kuo and Wei 2024)
Processed animal proteins (meta-analysis)	Broilers	Improved average daily gain in starter and grower phases	(Njeri et al. 2024)
MBM	Broilers	Maintained performance in low-protein diets and reduced nitrogen excretion	(Askri et al. 2025)
Insect larvae meal	Broilers	Phased inclusion improved growth and reduced inflammatory response	(Chen et al. 2025)

## Safety, regulatory frameworks, and analytical detection methods for PAPs

Safety considerations and regulatory controls form one of the most influential dimensions of the discussion surrounding processed animal proteins. Poultry nutritionists evaluate PAPs not only for their nutrient density and digestibility but also for the potential risks they may pose if improperly produced, contaminated, adulterated, or misidentified. The reintroduction of PAPs into animal feeding systems, as is occurring in several regions, is only possible because analytical methods have matured enough to monitor species origin, tissue composition, and contamination with high reliability. Historically, the most important regulatory driver was the need to prevent transmissible spongiform encephalopathies. The BSE crisis in the 1990s generated sweeping bans on the use of animal-origin proteins in livestock feeds. In the European Union, this took the form of the Animal By-Products Regulation and the so-called “total feed ban,” which prohibited feeding PAPs to food-producing ruminants and severely limited their use in other monogastric species. As Fumière et al. (2009) explain, this regulatory atmosphere drove the development of multiple analytical detection technologies designed to recognize prohibited animal proteins in feed matrices. These tools were necessary not only for enforcement but also for rebuilding confidence that scientifically verified controls could prevent cross-contamination.

Microscopy was one of the earliest and most widely used detection methods. It remains an official control tool in the EU, described extensively by Fumière et al. (2009). Under microscopy, analysts look for characteristic bone fragments, muscle fibers, or keratin structures that indicate the presence of animal proteins. Although microscopy is robust and relatively inexpensive, its sensitivity depends on operator expertise, and distinguishing between species can be difficult. Microscopy also struggles when proteins are thermally damaged, as heat processing can distort or destroy identifiable structures. These limitations eventually drove the adoption of molecular techniques capable of offering higher specificity. PCR–based DNA detection became one such tool. Studies like those referenced by Axmann et al. (2015) show that PCR assays can detect species-specific DNA even at low concentrations. However, DNA degradation during rendering complicates interpretation. Fumière et al. (2009) highlight that heavily processed PAPs may produce false negatives because DNA fragments become too short or chemically damaged. Conversely, some feeds containing dairy products or trace contamination from permitted sources may yield false positives. The sensitivity of PCR to extremely small quantities of DNA is both a strength and a challenge, requiring careful validation and routine use of controls.

Advances in proteomics have addressed many of these issues. Mass spectrometry–based approaches, such as those developed by Marbaix et al. (2016) and later refined by Steinhilber et al. (2019), identify species- or tissue-specific peptide markers that persist even after harsh thermal processing. These peptide markers provide a more stable biochemical signature than DNA in many PAP matrices. Steinhilber et al. (2019) demonstrated the value of targeted mass spectrometry in quantifying banned ruminant proteins within complex feed mixes and PAP samples. Because peptides survive where DNA does not, proteomic detection has emerged as one of the most reliable techniques for enforcement of species-exclusion rules. Complementary to molecular tools, spectroscopic methods play a growing role in PAP classification and fraud detection. De la Haba et al. (2009) and Pu et al. (2014) showed that near-infrared spectroscopy and FT-IR spectroscopy can differentiate PAP types based on characteristic lipid, protein, and mineral signatures. These tools allow rapid screening without labor-intensive sample preparation. Their main strengths lie in authentication and quality control rather than species identification, but they contribute significantly to routine industrial monitoring. A rendering plant or feed mill can use spectroscopy to verify consistency across batches, detect unusual mineral loads, or identify deviations in fat composition that could signal contamination or processing defects.

Safety concerns extend beyond species identification. Microbiological hazards represent another critical area of scrutiny. Traditional PAPs produced via rendering typically reach temperatures high enough to destroy vegetative pathogens, but spore-forming bacteria are more resilient. The rise of insect-derived PAPs brought renewed attention to microbial risks because insects reared on variable organic substrates can carry *Clostridium* species or other spore-forming microorganisms. Grenda et al. (2021) documented the presence of *Clostridium* strains in certain insect meals, emphasizing that drying and heating regimes must be optimized to ensure adequate microbial reduction. Although insect PAPs offer favorable nutritional profiles as described by Józefiak et al. (2016), their microbiological safety depends on rigorous adherence to processing standards, making hazard analysis and preventive controls essential. Antibiotic residues constitute another relevant risk category. Morello et al. (2021) conducted a targeted investigation into tetracycline residues in a broad sample of PAPs and found that a nontrivial portion contained measurable residues. Even more concerning, some samples retained detectable antimicrobial activity despite undergoing rendering. The persistence of antimicrobial residues has implications for antimicrobial resistance, regulatory compliance, and gut microbiome stability in poultry. This study underscores that safety risks in PAPs are not limited to microbiological contaminants or prohibited proteins but include chemical residues that require separate monitoring. Residue persistence may also reflect upstream issues in animal production systems, further illustrating that PAP safety reflects the entire supply chain rather than processing alone.

Work by the European Food Safety Authority (EFSA), including the comprehensive risk assessment by Adkin et al. (2011) contributed to refining EU rules to allow certain non-ruminant PAPs to be reintroduced into aquaculture and later into monogastric livestock diets under strict conditions. In an economic and policy analysis, it was pointed out that reintroducing PAPs into monogastric diets could reduce reliance on soybean meal and improve sustainability, provided that analytical detection systems ensured species segregation (Veldkamp 2012). The EFSA guidance emphasizes that cross-contamination, particularly between ruminant- and non-ruminant-derived PAPs, remains the critical risk. Analytical advances have therefore become the backbone of regulatory confidence that PAP use can expand safely. While the European Union has developed one of the most comprehensive regulatory frameworks governing PAP use, regulatory approaches differ substantially across other regions. In the United States, PAPs derived from approved animal by-products have long been utilized in poultry feeds under oversight by the Food and Drug Administration (FDA) and the Association of American Feed Control Officials (AAFCO), with restrictions focused primarily on preventing BSE transmission through ruminant feed chains. Canada follows a similar risk-based approach, while countries such as Brazil and Australia permit the use of selected non-ruminant PAPs under established rendering, traceability, and feed safety requirements. In contrast, implementation in many developing countries remains more variable and may be constrained by limited analytical infrastructure, inconsistent traceability systems, and differing levels of regulatory enforcement. Consequently, the practical adoption of PAPs depends not only on nutritional value but also on regional regulatory capacity, surveillance systems, and consumer acceptance. Monitoring frameworks now typically require a combination of approaches. Microscopy detects morphological structures; PCR tests allow species-level DNA identification; proteomics confirms peptide markers; and spectroscopy supports authentication and compositional verification. Together, these methods form a multilayered safeguards system. The studies, especially those by Fumière et al. (2009), Marbaix et al. (2016), and Steinhilber et al. (2019), show how such layered systems dramatically reduce the odds of both false negatives and false positives, enabling regulators to distinguish permissible PAP types from prohibited ones. This ensures that monogastric livestock like poultry can benefit nutritionally from PAPs inclusion while regulators retain confidence in safeguards preventing intra-species recycling.

Regulatory monitoring also addresses processing adequacy. Bellagamba et al. (2015) showed that amino acid racemization, particularly the presence of D-aspartic acid, can serve as a marker of excessive heat treatment. This insight has two regulatory implications. First, racemization helps ensure compliance with processing standards intended to guarantee microbial safety. Second, it provides quality control data for nutritionists evaluating nutrient availability. Although racemization itself is not a direct safety hazard, it indicates processing intensity, which can correlate with reduced nutritional quality, making it a dual-purpose indicator for both safety enforcement and formulation decisions. The safety considerations outlined in the literature also shape how PAPs can be used in poultry diets. Van Krimpen et al. (2018) stressed that formulating with PAPs requires not only amino acid balancing but also strict adherence to the rules that prevent co-mingling of species. Their broiler studies used PAPs that were fully compliant with EU regulations, demonstrating that safe use is entirely feasible when quality assurance systems are strong. Veldkamp et al. (2010) examined the welfare implications of PAP use and reported no adverse effects, further supporting that compliant PAPs pose no inherent welfare risks.

Some PAPs also pose potential issues related to mineral load. MBM, for instance, can have very high ash concentrations. While this is not a biological hazard in the classical sense, excessive mineral levels can distort calcium-to-phosphorus ratios, affect skeletal development, and alter digestive conditions in the gut. Janmohammadi et al. (2009) observed poorer performance in birds fed PBM with excessively high ash content. Therefore, regulatory and quality control frameworks commonly impose upper limits on ash content or require labeling clarity to ensure appropriate formulation decisions. The overall safety narrative surrounding PAPs reflects a balance between opportunity and oversight. The nutrient value of PAPs is evident, as demonstrated across the studies in digestibility, performance, and compositional research. But safety, in regulatory terms, depends on the ability to identify species origin, monitor contamination risks, track chemical residues, and enforce processing standards. Analytical science serves as the foundational tool enabling this balance. Without reliable detection of prohibited tissues, regulators would be unable to allow PAPs in poultry feeds. Without reliable detection of antibiotic residues or microbial hazards, nutritionists would be unable to incorporate PAPs with confidence.

What emerges from the combined literature is a picture of a well-regulated, analytically supported system in which PAPs can be used safely and effectively. The safeguards are not incidental; they are the product of decades of method development, from microscopy to PCR, from peptide marker mass spectrometry to rapid spectroscopic fingerprinting. These tools enable the poultry industry to reintroduce PAPs in ways that protect public health, animal health, and consumer confidence (Fumière et al. 2009; Marbaix et al. 2016; Steinhilber et al. 2019; Grenda et al. 2021; Morello et al. 2021). As PAP use expands to include insect proteins and hydrolysis-based ingredients, regulatory systems will continue to adapt. Emerging hazards associated with new PAP types, such as substrate-dependent contaminants or alternative processing-induced changes, will require new analytical markers and adapted control frameworks. But the foundation established by the existing body of research gives a strong basis for ensuring that these new PAPs enter feed systems safely.

## Practical formulation strategies, integration into feeding programs, and future directions

Formulating poultry diets with processed animal proteins requires more than substituting one protein source for another. PAPs differ from plant proteins in nutrient density, amino acid balance, metabolizable energy, mineral content, and functional bioactivity. These differences can offer advantages when properly incorporated, but they also require precise formulation strategies that account for the complexity of PAP composition and digestibility.

A central principle that recurs across literature is the need to formulate diets based on digestible amino acids rather than crude protein alone. Secondly, processing plays a vital role in the nutritional value of PAPs. For example, a PBM processed under control conditions may deliver high SID for lysine and methionine, while an over-processed batch with similar crude protein levels could provide far less available amino acid content. This means nutritionists must rely on laboratory-tested digestibility coefficients and, when possible, supplier-specific nutrient matrices, instead of generic values for meat meal, BM, or FM.

One of the most important formulation considerations is balancing mineral contributions. MBM and many PBM products carry significant calcium and phosphorus loads. If these mineral levels are not accounted for, diets can exceed optimal Ca: P ratios. Over-supplying calcium can adversely affect the digestibility of other nutrients and contribute to skeletal issues, while under-supplying available phosphorus leads to poor bone mineralization. In practice, PAPs often reduce the need for expensive inorganic phosphate supplementation, but the mineral contribution must be calibrated carefully. Overly high ash levels, as the studies show, can depress performance by diluting digestible nutrients and disrupting normal physiology.

HFM represents another formulation challenge. Its high sulfur amino acid content makes it potentially valuable, but because digestibility depends heavily on processing quality, nutritionists must incorporate HFM only when verified solubility and digestibility data are available. It appears that an under-processed HFM meal cannot replace conventional protein sources effectively. This means FM should be introduced in controlled amounts, usually below 5% of the diet dry matter, until consistency and quality are assured.

BM and PPs offer different formulation opportunities. BM is extremely rich in lysine and carries high digestibility when gently processed. PPs supply functional components that improve gut development and immune resilience in early life stages. For this reason, blood-derived PAPs can be included in pre-starter diets for chicks, where maximizing early feed efficiency and gut stability is a priority. These functional benefits reduce the need for certain feed additives in early diets, such as some gut-health supplements, because plasma fractions already contribute immunological support.

Insect-based PAPs present formulation opportunities but also uncertainties due to ongoing standardization. BSFL meals have favorable amino acid profiles and can serve as high-quality protein sources. However, batch consistency can vary because nutrient composition depends on the insects’ rearing substrate. Microbial hazards vary across insect PAPs and depend on processing rigor. Formulators, therefore, must treat insect PAPs as evolving ingredients: promising, but currently best incorporated with conservative inclusion rates and regular nutrient and microbiological testing. Enzymatically hydrolyzed insect proteins may offer more predictable digestibility and could play an increasing role in future poultry nutrition where peptide-rich protein sources are desired.

Functional hydrolysates made from rendered by-products or meat processing wastewater offer yet another direction for PAP integration. Bacterial fermentation and enzymatic hydrolysis of meat industry effluents can yield highly digestible peptide fractions that support strong feed efficiency and growth in broilers. These hydrolysates have solubility and digestibility advantages compared with intact proteins, making them suitable for inclusion in diets where rapid absorption of amino acids is beneficial, such as stress recovery diets or pre-starter feeds. Antimicrobial activity or residue risks, however, must be monitored.

Quality assurance is key in all formulation strategies. The analytical tools described by previous researchers (Fumière et al. 2009; Marbaix et al. 2016; Steinhilber et al. 2019; Grenda et al. 2021; Morello et al. 2021), are not merely regulatory tools; they are practical aids for nutritionists who need to verify ingredient identity, predict nutrient composition, detect adulteration, and ensure compliance with species-exclusion rules. Spectroscopic fingerprinting helps identify deviations in fat or mineral composition. PCR and proteomic peptide markers confirm species purity. Racemization markers provide indicators of overprocessing, which alert nutritionists to potential digestibility losses. Collectively, these tools help maintain formulation accuracy and protect performance outcomes.

Economics also drives practical PAP use. Reintroducing PAPs into poultry diets can reduce cost burdens associated with imported soybean meal. The economic benefit depends on reliable nutrient values; however, inconsistent PAP quality erodes cost-effectiveness because it increases the risk of underperformance and forces the nutritionist to incorporate safety margins. This tension between economic opportunity and variability reinforces the need for supplier certification programs, standard processing protocols, and laboratory-based nutrient profiling.

Integration of PAPs into commercial feeding programs also requires consideration of how diet processing interacts with ingredient characteristics. Pelleting temperatures, for instance, can influence protein integrity. If a diet contains PAPs already exposed to high heat loading during rendering, additional heat from pelleting may exacerbate amino acid damage. Nutritionists, therefore, must coordinate with feed mills to ensure pelleting temperatures are not excessive when high-PAP diets are produced. Fat applied during coating or post-pelleting can help protect some heat-labile compounds, but these details require careful coordination.

Looking ahead, the literature suggests several future directions for PAP use in poultry nutrition. One avenue involves refining hydrolysis technologies to increase digestibility and reduce peptide variability. Both feather-derived PAPs and insect PAPs stand to benefit from more precise hydrolysis methods that target specific peptide lengths, enhancing amino acid uptake. Another direction involves combining PAPs with novel feed enzymes. For instance, protease supplementation could further increase the digestibility of collagen-rich proteins, especially in partially hydrolyzed meals.

A third direction comes from sustainability pressures. As global interest grows in circular nutrition systems, PAPs derived from waste streams, such as the hydrolysates, could expand considerably. Similarly, insect PAPs produced on waste substrates offer environmental advantages that align with increasing industry focus on climate impact mitigation.

Regulatory evolution will also shape future applications. The risks of cross-species exposure and recommended species-segregation approaches that allow monogastric animals to use non-ruminant PAPs safely should be evaluated and researched. As detection technologies continue to advance, regulators may consider expanding the permitted uses of PAPs, provided that analytical safeguards remain strong enough to prevent prohibited recycling. Such expansions could improve access to animal protein sources across global markets, especially in regions where soybean meal is costly or limited.

In many ways, the future of PAPs’ use depends on the same factors that determine their present value: digestibility, safety, processing quality, and economic viability. As long as nutritionists and regulators continue to apply rigorous analytical and processing standards, PAPs will remain a cornerstone of sustainable, efficient poultry nutrition. They offer advantages, high-digestible amino acids, functional protein fractions, and efficient nutrient recovery that plant proteins alone cannot always supply. With improved standardization, expanding research, and ongoing policy support, PAPs are positioned to contribute even more substantially to poultry nutrition programs in the years ahead.

## Conclusion

Processed animal proteins represent a diverse group of nutrient-dense ingredients that can contribute meaningfully to poultry diets when their composition, processing, and safety are well controlled. The evidence across compositional analyses, digestibility studies, and production trials shows that PAPs can supply highly digestible amino acids, functional peptides, and valuable minerals while reducing dependence on imported plant proteins. Their nutritional performance is closely tied to processing quality: controlled rendering, spray-drying, or enzymatic hydrolysis preserve amino acid integrity and deliver strong digestible nutrient values, whereas excessive heat exposure reduces lysine availability and increases racemization. Blood-derived products, hydrolyzed feather proteins, PBM, and several insect PAPs consistently support growth and feed efficiency when diets are formulated using digestible amino acids and balanced for minerals.

Safety and regulatory oversight remain essential for maximizing the benefits of PAPs. Advances in microscopy, PCR, peptide-marker mass spectrometry, and spectroscopic tools now allow reliable detection of species origin, processing adequacy, and contamination risks, enabling controlled reintroduction of non-ruminant PAPs into monogastric diets. Studies also highlight the importance of monitoring for chemical residues and microbial hazards, especially in novel PAPs such as insect meals. When ingredient identity is verified and nutrient values are accurately characterized, PAPs function as safe, sustainable, and cost-effective protein sources. Continued refinement in processing technologies, analytical methods, and quality standards will further strengthen their role in modern poultry nutrition.

## Data Availability

Not applicable.
